# Generation of genetically-engineered animals using engineered endonucleases

**DOI:** 10.1007/s12272-018-1037-z

**Published:** 2018-05-17

**Authors:** Jong Geol Lee, Young Hoon Sung, In-Jeoung Baek

**Affiliations:** 1 ConveRgence mEDIcine research cenTer (CREDIT), Asan Institute for Life Sciences, Asan Medical Center, Seoul, Republic of Korea; 2 College of Veterinary Medicine, Chungbuk National University, Cheongju, Republic of Korea; 3 Department of Convergence Medicine, ConveRgence mEDIcine research cenTer (CREDIT), Asan Institute for Life Sciences, Asan Medical Center, University of Ulsan College of Medicine, Seoul, Republic of Korea

**Keywords:** Genome editing, Engineered endonuclease, ZFN, TALEN, CRISPR

## Abstract

The key to successful drug discovery and development is to find the most suitable animal model of human diseases for the preclinical studies. The recent emergence of engineered endonucleases is allowing for efficient and precise genome editing, which can be used to develop potentially useful animal models for human diseases. In particular, zinc finger nucleases, transcription activator-like effector nucleases, and the clustered regularly interspaced short palindromic repeat systems are revolutionizing the generation of diverse genetically-engineered experimental animals including mice, rats, rabbits, dogs, pigs, and even non-human primates that are commonly used for preclinical studies of the drug discovery. Here, we describe recent advances in engineered endonucleases and their application in various laboratory animals. We also discuss the importance of genome editing in animal models for more closely mimicking human diseases.

## Introduction

In the fields of drug discovery and development, in vivo experiments using appropriate animal models are indispensable for successful clinical translation, which can lead to the development of preventatives or treatments for human diseases. Additionally, studies adopting genetically-engineered animals can also provide us the important clues for the underlying mechanisms that make us to understand the pathology of the disease.

Genome editing refers to the manipulation of specific gene loci with engineered endonucleases in cultured cells or living organisms to modify the genome (Cong et al. 2013; Gaj et al. 2013; Shao et al. 2016). The key step in genome editing is the induction of site-specific double-strand breaks (DSBs) by engineered endonucleases that are subsequently corrected by one of two competing DNA repair pathways, non-homologous end-joining (NHEJ) and homology-directed repair (HDR) (Sander and Joung 2014): NHEJ is characterized by the direct ligation of two DNA DSB ends frequently introducing unpredictable patterns of insertions and/or deletions (in-dels) leading to gene disruption or “knockout” through frameshift mutation, while HDR is an error-free mechanism that can be used to induce sequence-defined mutations or to insert a desired sequence at the targeted locus (Ceccaldi et al. 2016; Li et al. 2017).

Recent advances in genome editing technologies reflect the rapid development of engineered endonucleases including zinc finger nucleases (ZFNs), transcription activator-like effector nucleases (TALENs), and clustered regularly interspaced short palindromic repeat (CRISPR) systems, and now we can rapidly generate disease-associated animal models for a wide range of species (Dow 2015; Wang and Qi 2016). The engineered endonuclease-mediated genome editing approach has been established in many species, particularly in laboratory animals such as mice (Carbery et al. 2010; Meyer et al. 2010; Sung et al. 2013; Wang et al. 2013), rats (Geurts et al. 2009; Tesson et al. 2011; Li et al. 2013a, b), rabbits (Flisikowska et al. 2011; Song et al. 2013; Yang et al. 2014), dogs (Zou et al. 2015), pigs (Whyte et al. 2011; Carlson et al. 2012; Hai et al. 2014), and even non-human primates (Sato et al. 2016; Niu et al. 2014; Liu et al. 2014). The expanded application of genome editing to generate transgenic animals beyond mice will be advantageous in biomedical research because of its potential to accelerate the development of new therapeutic strategies (Wang and Qi 2016).

Here, we summarize the practical applications of three major engineered endonucleases—ZFN, TALEN, and CRISPR—that have been successfully used to edit genomes, with an emphasis on those of laboratory animals. In addition, we describe their advantages, disadvantages, and recent advances in genome editing. We hope to inform readers which tools can be useful options for desired tasks in desired laboratory animals.

## Engineered endonucleases frequently used in generating genetically-engineered animals

An endonuclease must exhibit two characteristics to be useful for genome editing: (1) specific recognition of target sequences and (2) sufficient adaptability to target user-defined sequences (Urnov et al. 2010). The four genome-editing tools—ZFN, TALEN, and CRISPR/Cas including Cas9 and Cpf1—all satisfy these specifications, but there are some differences among them in terms of origin, structure, and action mechanism.

## Zinc finger nuclease

ZFN was first devised by Kim et al. in 1996 and was initially applied to the fruit fly for genome editing (Bibikova et al. 2002, 2003).

ZFN is composed of the DNA binding domain of zinc finger protein (ZFP) linked with the endonuclease domain of the FokI restriction enzyme. ZFN acts through DNA/protein recognition, as the ZFP region contains 3–6 tandem fingers, each of which recognize 3 bp of DNA (Miller et al. 1985; Wolfe et al. 2000). As FokI must dimerize to cleave DNA and this interaction is weak (Vanamee et al. 2001), ZFN should be designed as a pair, one on the forward strand and the other on the reverse strand, to permit the correct orientation and appropriate spacing for FokI dimerization. Once ZFP regions bind to either side of the target site (the total of both sides, 18–36 bp), the FokI domains dimerize and cleave the target DNA sequence (Urnov et al. 2010), generating a site-specific DSB that is subsequently repaired via the HDR or NHEJ pathway.

## Talen

TAL effector (TALE) represents the largest family of type III effector proteins from *Xanthomonas* spp., a group of gram-negative bacterial plant pathogens that was first discovered in 1989 (Bonas et al. 1989). Its capability to bind to DNA was first described in plants in 2007 (Romer et al. 2007; Kay et al. 2007), and the codes for recognizing the target DNA by TALE proteins were decrypted in 2009 (Moscou and Bogdanove 2009; Miller et al. 2011).

The DNA binding domain of naturally occurring TALE consists of 10–30 tandem repeats of the 34-amino acid module, which is highly conserved except for two hypervariable amino acid residues at positions 12 and 13, referred to as repeat-variable di-residue (RVD). The first base of the target recognized by an N-terminus region of TALE is generally specific for thymine, and the remaining bases are sequentially bound to RVD, in the manner where one type of RVD preferentially recognizes a specific nucleotide. Like ZFN, TALEN is generated by fusing the FokI endonuclease element to the engineered TALE-binding domain, and binds to the target sequence as dimers: each monomer binds to a “half-site” in the target and the FokI endonuclease domains dimerize to generate a DSB in the spacer sequence between the two half-sites.

## CRISPR/Cas9

The CRISPR/CRISPR-associated protein (Cas) system was first observed in prokaryotes that mediate a bacterial adaptive immune defense against viruses or invading nucleic acids in 2007 (Barrangou et al. 2007). It was revealed in 2012 that mature dual RNA (crRNA:tracrRNA), following co-processing of tracrRNA and pre-crRNA by RNaseIII, is sufficient for Cas9-catalyzed DNA cleavage in *Streptococcus pyogenes* (Jinek et al. 2012), and subsequently, first evidences of genome editing using the CRISPR/Cas9 system were reported in mouse and human cells in 2013 (Cong et al. 2013; Mali et al. 2013b).

The CRISPR/Cas system is characterized by incorporating fragments of invading nucleic acid as spacers into a host genome and in the case of later infection, using them as templates to generate small RNA molecules (crRNA) that are combined with Cas proteins into an effector complex to silence foreign nucleic acids (Makarova et al. 2011). According to the latest classification based on the configuration of their effector modules, the diverse CRISPR-Cas systems can be divided into two classes: (1) class 1 CRISPR systems, which utilize several Cas proteins and crRNA to form an effector complex that includes type I and type III CRISPR systems, and (2) class 2 CRISPR systems, which employ a large single-component Cas protein in conjunction with crRNAs to mediate interference. In particular, type II CRISPR systems only require Cas9 protein as an effector for DNA interference (Makarova et al. 2015).

In the CRISPR/Cas9 system, single guide RNA (sgRNA or gRNA) that is engineered as a complex of CRISPR RNA (crRNA) and trans-activating crRNA (tracrRNA) brings the endonuclease complex into the specific target site on the genome and then recruits Cas9 protein for precise DNA cleavage. sgRNA-guided target selection in the CRISPR/Cas system, particularly Cas9 from *S. pyongenes*, requires a G-rich (NGG) protospacer adjacent motif (PAM) sequence at the 3′-end of the target site, which determines Cas9 binding specificity to its target region. Once bound to the target sequence followed by PAM, Cas9 generates DSB 3–4 nucleotides upstream of the PAM site (Mali et al. 2013b). Table 1 summarizes the characteristics of ZFN, TALEN, and CRISPR/Cas9 in genome editing.

**Table 1:** Comparison of three engineered nucleases-ZFN, TALEN, and CRISPR/Cas9

	ZFN	TALEN	CRISPR/Cas9
Backbone origin	Highly prevalent in eukaryotes	Bacteria (*Xanthomonas* spp.)	Bacteria (*S. pyogenes*)
Specificity module	ZFP	TALE	sgRNA (crRNA + tracrRNA complex)
Cleavage module	FokI	FokI	Cas9
Target site	18–36 bp (3 nt per zinc finger module)	30–40 bp (1 nt per RVD; TALE binding sites should start with a T)	20 bp + PAM (NGG) sequence (Cas9 binding sites should end with G-rich PAM)
Working mechanism	DNA/protein recognition, DSB, and its repair pathway	DNA/protein recognition, DSB, and its repair pathway	DNA/RNA recognition, DSB, and its repair pathway
Reprogramming efficiency	Relatively low	Relatively low	High (easier to design, faster to synthesize, and cost-effective; furthermore, multiplex genome editing is available)

*ZFN* zinc finger nuclease, *ZFP* zinc finger protein, *TALE* transcription activator-like effector, *TALEN* TALE nuclease, *RVD* repeat-variable di-residue, *CRISPR* clustered regularly interspaced short palindromic repeat, *Cas9* CRISPR-associated enzyme 9, *sgRNA* single guide RNA, *crRNA* CRISPR RNA, *tracrRNA* trans-activating crRNA, *PAM* protospacer adjacent motif

## CRISPR/Cpf1

Among class 2 CRISPR systems, a new type V CRISPR-Cas endonuclease, Cpf1 was first identified in *Francisella* and later in other bacteria *Prevotella* (CRISPR from *Prevotella* and *Francisella* 1) (Schunder et al. 2013; Vestergaard et al. 2014; Makarova et al. 2015). The CRISPR/Cpf1 system was first applied as a genome editing tool in human cells in 2016, and it has three main distinct features from Cas9 (Zetsche et al. 2015): (1) tracrRNA is not required and thus the crRNA of Cpf1 is notably shorter than the sgRNA of Cas9. (2) sgRNA-Cpf1 complexes target DNA to produce DSB distal to a 5′-end T-rich PAM sequence, in contrast to Cas9, which produces DSB proximal to the 3′-end G-rich PAM site. (3) Cpf1 produces staggered DSB with a 4 or 5-nucleotide 5′-overhang (sticky end cut), whereas Cas9 cuts both strands in a DNA molecule at the same position (blunt end cut). Table 2 summarizes and compares the characteristics of Cas9 and Cpf1.

**Table 2:** Comparison of the characteristics of CRISPR/Cas9 and Cpf1

	CRISPR/Cas9	CRISPR/Cpf1
Backbone origin	*Streptococcus pyogenes* (*SpCas9*)	*Francisella novicida* (*FnCpf1*), *Acidaminococcus* sp. *BV3L6* (*AsCpf1*), *Lachnospiraceae bacterium* (*LbCpf1*)
Structure of sgRNA	crRNA + tracrRNA	crRNA
Nuclease domain	RuvC-like + HNH	RuvC-like
PAM site	G-rich (5′-NGG)	T-rich (3′-NTT)
Cutting mechanism	Blunt cut 3 nt upstream of the PAM (close to PAM)	Staggered cut (with 4–5 nt overhang) 17 nt downstream of the PAM (far from PAM)

*CRISPR* clustered regularly interspaced short palindromic repeat, *Cas9* CRISPR-associated enzyme 9, *sgRNA* single guide RNA, *crRNA* CRISPR RNA, *tracrRNA* trans-activating crRNA, *PAM* protospacer adjacent motif

## Specificity of engineered endonuclease

The most important hurdle to surmount in engineered endonuclease-mediated genome editing is its specificity and the off-target issue; the higher the specificity of the engineered endonucleases, the lower their off-target cleavage and hence their toxicity.

In ZFNs, the efficacy is largely dependent on the specificity of ZFPs. One way of increasing its specificity is to assemble the ZFP with an increased number of zinc finger modules to create a longer DNA recognition site, but this is not always sufficient. Another complementary approach is to design ZFN in a way such that the dimerization of the FokI cleavage domain occurs in the formation of a heterodimer rather than a homodimer, which will actively cleave only at specific heterodimer binding sites rather than at the homodimer or unintended binding sites (obligate heterodimerization) (Miller et al. 2007; Szczepek et al. 2007). The combination of both approaches has been successful with high specificity in zebrafish embryos (Doyon et al. 2008), rat embryos (Geurts et al. 2009; Mashimo et al. 2010), and mammalian cells (Urnov et al. 2005; Lombardo et al. 2007; Perez et al. 2008; Hockemeyer et al. 2009), and there were also some improvements in the obligate heterodimerization strategy for ZFNs; for example, to abolish non-specific protein binding to the DNA backbone via amino acid substitutions (Ramalingam et al. 2011). These strategies are also beneficial for enhancing the specificity of TALENs (Huang et al. 2011; Hockemeyer et al. 2011; Cade et al. 2012).

The off-target issue is also the biggest concern in the CRISPR/Cas9 system (Fu et al. 2013; Hsu et al. 2013; Mali et al. 2013a; Pattanayak et al. 2013) and various efforts have been made to improve its specificity. Two methods that use distinct types of Cas9 pairs have been developed to reduce off-target effects in the CRISPR/Cas9 system: (1) A mutant version of Cas9 “nickase,” in which one of either the Cas9 endonuclease domain histidine-asparagine-histidine (HNH) or RNase H-like fold (RuvC) is inactivated, can only introduce single-strand DNA breaks rather than DSB. Pairing the two nickase with their sgRNAs, allows a DSB with a 5′-overhang to be introduced at the target site, while single-strand nick at the off-target site would be fixed (Ran et al. 2013). (2) Another strategy to reduce off-target effects is to use of a pair of proteins, in which catalytically dead Cas9 (dCas9) is fused with the FokI domain. When the two FokI-dCas9 pairs are guided by two sgRNAs and subsequently positioned on both directions of the DNA strands, FokI endonuclease domains dimerize to generate a DSB in the on-target site (Tsai et al. 2014; Guilinger et al. 2014). The applications of the two above methods show drastically increased on-target specificity with reduced unexpected mutations in human cells, but it remains to be elucidated whether they work in vivo. Of course, it is always necessary to backcross mutant lines with multiple generations to remove any off-target mutations and/or verify the phenotypes with more than two independent lines.

## Genome editing using engineered endonucleases in laboratory animals

**Fig. 1:**
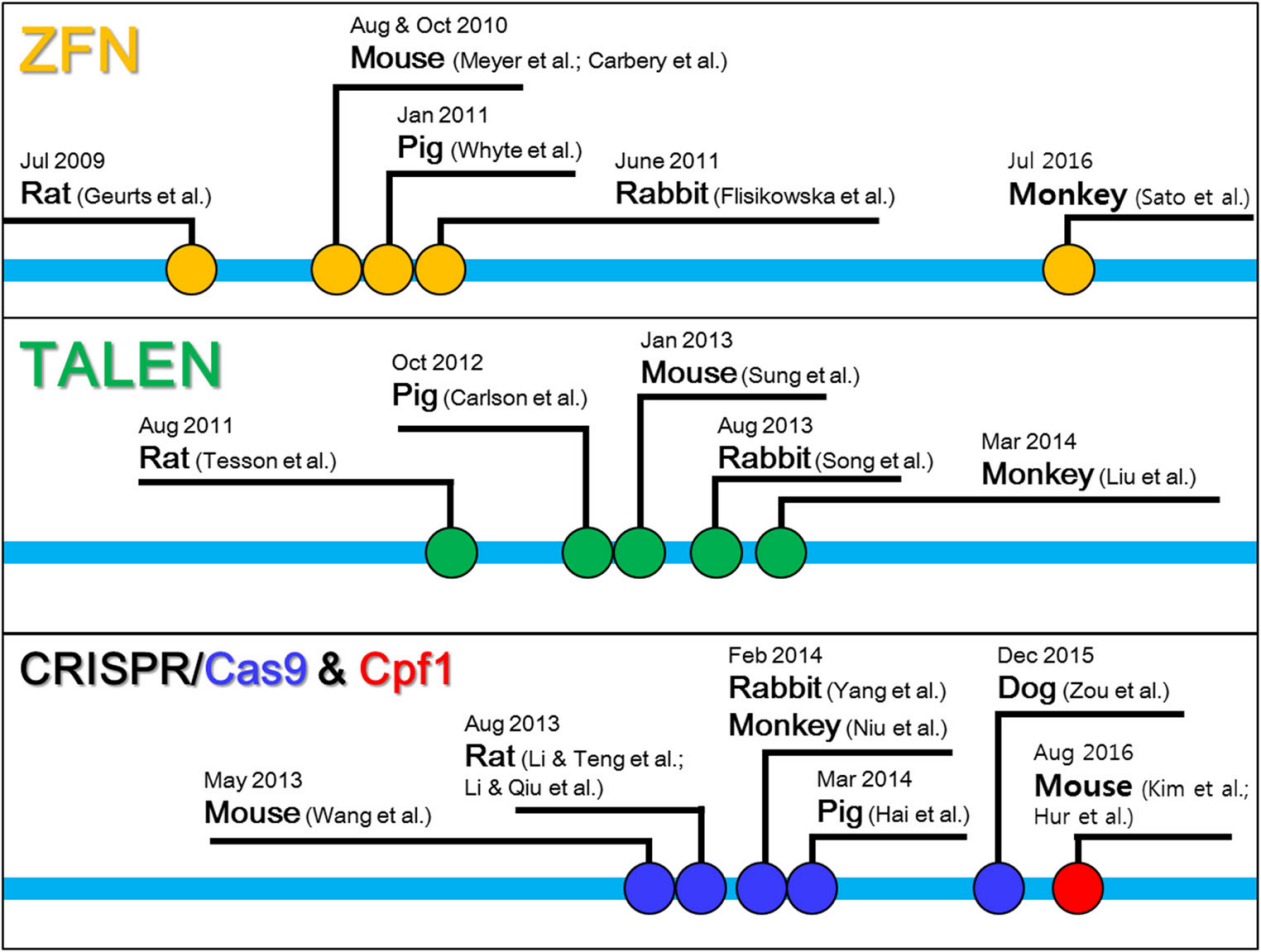
The timeline for the first applications of engineered nuclease technologies in laboratory animals. The time points when studies that describe the first applications of zinc finger nuclease (ZFN), transcription activator-like effector nuclease (TALEN), and clustered regularly interspaced short palindromic repeat (CRISPR) system including CRISPR-associated enzyme 9 (Cas9) and CRISPR from *Prevotella* and *Francisella* 1 (Cpf1) for genome editing in various laboratory animals were published are marked with colored circles (ZFN, yellow; TALEN, green; Cas9, blue; Cpf1, red)

Numerous model organisms have been developed and are now available to researchers, and mammalian models are now extensively used for studying basic biology and pathophysiology of human diseases and the development of novel therapeutics (von Horsten et al. 2003; Golding et al. 2006; Yang et al. 2008; Gilley et al. 2011; Hauschild et al. 2011; Zschemisch et al. 2012; Chan 2013). Studies that describe the first applications of ZFN, TALEN, and CRISPR in laboratory animals are summarized in Fig. 1.

## Mouse

The mouse is the best-known and has been the most widely used mammalian model over the past century, particularly in the field of drug discovery and development, because therapeutic agents can be tested using mice in expeditious, cost-effective, and ethical manners. In genome engineering, it is an ideal animal in many aspects: (1) relatively low cost to maintain, (2) the short life-span beneficial to breed enough animals quickly and also suitable for the genetic studies, (3) it can develop various human-like diseases with wild-type and genetically-engineered animals, (4) 99% of its genes are shared with humans (Boguski 2002), and (5) many genetic resources have been developed and are now publicly available (Paigen 1995; Eppig 2017).

In the past, traditional genome editing in mice was only dependent on the HDR technique in embryonic stem (ES) cells (Capecchi 1989). However, this strategy is characterized by multiple complicated steps: maintenance of ES cells in an undifferentiated state, construction of the targeting vector that should be electroporated into ES cells, positive and negative selections to enrich homologous recombinant ES cells, cloning and screening, expansion of correctly targeted ES cell clones, and the production of chimeric mice with germline transmission abilities. This method requires an enormous amount of time- and effort-consuming work, with no guarantee of success in every step.

Engineered endonuclease dramatically simplified the complicated processes for generating genetically-engineered mice (GEM), and was selected by *Nature Methods* as the “Method of the Year 2011” (2012). After the initial observation that genome editing using ZFN became applicable in the fruit fly (Bibikova et al. 2002, 2003), ZFN-based genome editing has been applied to both in vitro and in vivo models of various species, including CHO cells (epithelial cell lines derived from the ovary of the Chinese hamster) (Cost et al. 2010; Santiago et al. 2008), plant tobacco (Cai et al. 2009), zebrafish (Doyon et al. 2008; Meng et al. 2008), rat (Geurts et al. 2009), and mouse ES cells (Goldberg et al. 2010).

ZFN technology was first applied in mice by two groups in 2010: (1) Meyer et al. (2010) described how ZFN can improve HDR efficiency of the targeting vector into target site fertilized mouse embryos; and (2) Carbery et al. (2010) reported that gene disruption using ZFN can be achieved through NHEJ event. Through these studies, it became obvious that (1) ZFN can be applied and replace mouse ES cells to generate genetically-engineered mice and (2) rather, engineered endonucleases must be advantageous over the classical gene targeting in moue ES cells as genome editing can be achieved by the single step injection of ZFN in a strain (or genetic background)-independent manner.

In 2013, another FokI-based engineered endonuclease called TALEN was revealed to have specific gene targeting ability to produce knockout mice (Sung et al. 2013), and Wang et al. (2013) reported that CRISPR/Cas9 was also useful for genome editing in mice and that co-injection of multiple guide RNAs could induce multiple mutations in mouse genome, simultaneously. Moreover, it is recently revealed that Cpf1 can be a useful genome editing approach in mammals including mouse (Kim et al. 2016; Hur et al. 2016) and rat (data not yet published), but more researches into this next-generation engineered endonuclease are in progress to explore its application in a range of another mammals.

## Rat

The rat is a more widely used model for studying human normal physiology or diseases and for testing drug efficacy or toxicity prior to clinical trials in humans (Jacob 1999; Jacob and Kwitek 2002; Aitman et al. 2008). As an animal model for human diseases, the rat model provides many advantages over mouse: (1) its larger size compared to mouse provides easier handling and surgery, a larger sample volume, and high-resolution imaging, (2) the rich behavioral profile in rat is superior to that of mouse, particularly for learning and memory (Whishaw 1995; Whishaw and Tomie 1996), addiction (Jupp et al. 2013), and juvenile play (Hamilton et al. 2014), which is advantageous in neuroscience research, and (3) most importantly, rat is more translational than mouse due to its physiological similarity to human (Aitman et al. 2016).

Before the emergence of ES cell-mediated gene targeting, mutant rat strains were generated by random mutagenesis using a chemical mutagen N-ethyl-N-nitrosourea (ENU) or by introducing a *sleeping beauty* transposon (Zan et al. 2003; Izsvak et al. 2010). As the mutant strains were identified by phenotype-driven screening, these strategies require large populations of rats and were costly and time-consuming processes of high-throughput screening. For the gene targeting, several methodological attempts have been made to establish authentic rat ES cells, and a culture system optimized for maintaining rat ES cells was finally developed (Buehr et al. 2008; Li et al. 2008), leading to the production of p53 KO rats (Tong et al. 2010). However, as the rat ES cells seems to be less robust than mouse ES cell and now plenty of mutant mouse resources publicly available (e.g., International Mouse Phenotyping Consortium or IMPC), most of in vivo studies employing mutant animal models are adopting mouse models.

Considering the importance and the difficulties of ES cell-based genome editing, it is an inevitable consequence that the rat is the first mammal species applied for genome editing with ZFN (Geurts et al. 2009) and TALEN (Tesson et al. 2011). Pronuclear or intracytoplasmic microinjection of ZFN or TALEN-encoding DNA or mRNA into fertilized rat eggs results in targeted gene knockout with a high efficiency. The CRISPR/Cas9 system was also proved to be adaptable for rat genome editing, and in particular, the co-injection of two or more guide RNAs with Cas9 mRNA led to multiple disruptions simultaneously in the rat genome (Li et al. 2013a, b).

## Rabbit

The rabbit has been used for a long time in experimental research such as the production of antibodies or recombinant proteins and various toxicological studies for non-clinical safety studies, and was in fact the first animal model used for human atherosclerosis more than a century ago. Rabbit models for human disease have been developed to study lipid metabolism, atherosclerosis, osteoarthritis (Fan and Watanabe 2003; Martinez-Calatrava et al. 2010), and eye researches (Kang and Grossniklaus 2011; Zernii et al. 2016).

Regardless of the demand for genetically-modified rabbit, naturally occurring or spontaneous mutant strains were only available (Bosze and Houdebine 2006). Since the first transgenic rabbit was produced through the pronuclear microinjection of a DNA construct (Hammer et al. 1985), some methodological improvements have been reported for rabbit transgenesis (Viglietta et al. 1997; Besenfelder et al. 1998); however, it is still characterized by too-low transgenic efficiency (Houdebine and Fan 2009; Bosze et al. 2016). As the lack of fully functional rabbit ES cells was not produced (Honda et al. 2013; Honsho et al. 2015) and somatic cell nuclear transfer (SCNT) technologies are not established in rabbits (Zakhartchenko et al. 2011), still the targeted genome editing was not possible before the application of engineered endonucleases.

Those hurdles are now solved in rabbit by applying engineered endonucleases such as ZFN, TALEN, and the CRISPR/Cas9. In particular, in the last 5 years, studies using CRISPR/Cas9 for genome editing in rabbit have exceeded those of ZFN or TALEN quantitatively in spite of the relatively short history of the CRISPR system: there are only two or three studies of genetically-modified rabbit with ZFN (Flisikowska et al. 2011; Yang et al. 2013; Ji et al. 2015) or TALEN (Song et al. 2013, 2016a). However, we found 15 papers in the Medline applying CRISPR/Cas9 for genome editing in rabbit, and this number is continually increasing (Yang et al. 2014; Yan et al. 2014; Honda et al. 2015; Song et al. 2016a, b; Yuan et al. 2016; Lv et al. 2016; Yang et al. 2016; Sui et al. 2016; Guo et al. 2016; Song et al. 2017b; Yuan et al. 2017; Song et al. 2017a; Liu et al. 2018) due to its simplicity in design, high mutation efficiency, and the ability to simultaneously edit the genome of multiple genes, which seems superior to ZFN or TALEN in rabbit.

## Dog

Dogs are of great benefit in their service to humankind including companionship and working activities, and have been also used as models in biomedical research. The advantages of using dog as an animal model include its anatomy, physiology, genetics, behavior, and its relatively long lifespan and size, which more closely match humans than other species such as rodents (Chianese et al. 2011). Indeed, dogs have proven remarkable model systems to investigate various hereditary human diseases including leukocyte adhesion deficiency, Leber’s congenital amaurosis, Duchenne muscular dystrophy, and hemophilia A and B (Bauer and Hickstein 2000; Acland et al. 2001; Kinali et al. 2009; Margaritis 2010) because they have been selectively bred, which has resulted in a range of spontaneous and complex phenotypic variations that are often accompanied by undesired pathological genetic variations that were not observed in other species (Nowend et al. 2011; Switonski 2014). Many hereditary diseases in dogs naturally occur in very similar clinical manners to analogous human diseases (Shearin and Ostrander 2010).

Regardless of the importance of dogs in biomedical research and increasing demand for their genetic modification, studies into the manipulation of the dog genome are extremely limited in number. As compared to other mammals, dogs have certain unique species-specific characteristics that can be obstacles for artificial reproduction; for example, difficulty in synchronizing the reproduction stage between donor zygotes and recipient female dogs (Holst and Phemister 1971; Farstad 2000; Jang et al. 2007). These limit the application of dogs, particularly in the field of genome editing.

Finally, Zou et al. modified the dog genome successfully in 2015 using the CRISPR/Cas9 system, where the asynchronous reproduction stage in the donor and recipient was overcome via auto-transplantation of guide RNA/Cas9 mixture-injected zygotes into the same female dog (Zou et al. 2015). Therefore, the CRISPR/Cas9 system may be the primary choice for the generation of future novel dog models for biomedical research.

## Pig

Pigs are very similar to humans in terms of anatomy, genetics, and physiology: (1) In terms of anatomy, the similarities in the size and morphology of their internal organs to those of humans allows various surgical and non-surgical procedures in clinical settings such as catheterization, heart surgery, valve manipulation, endoscopy, and broncho-alveolar lavages, and thus pigs are frequently used as the general surgical model for both training and research over the last 20 years (Swindle et al. 2012). (2) The pig is phylogenetically closer to primates than rodents and thus the size and composition of the porcine genome are much more similar to those of humans. In particular, some inbred porcine strains with defined genetic background have been established, which enables us to obtain reproducible results (Kobayashi et al. 2012; Zhao et al. 2009). (3) In terms of physiology, the immune system in pigs is similar to that of humans, and their organs generally have functional features in common with humans (Swindle et al. 2012). In these regards, pigs have been considered as one of the major mammals in translational research, and considering their frequent application in studying the human diseases including Huntington’s disease (HD), Alzheimer’s disease (AD), retinitis pigmentosa, cystic fibrosis, cancer, and diabetes, the preclinical toxicological and efficacy testing of pharmaceuticals, and the xenotransplantation of pig organs to human (Aigner et al. 2010; Whyte and Prather 2011; Meurens et al. 2012), genetically-engineered pigs will be of great use in bio-medical studies.

The first transgenic pig was established by the microinjection of DNA construct into zygotes in 1985, and then various methodological procedures have been developed for efficient transgenesis in pigs, including sperm-mediated gene transfer (SMGT) (Lavitrano et al. 1997), intracytoplasmic sperm injection (ICSI)-mediated gene transfer (Kurome et al. 2006), and strategies employing viral vectors (Cabot et al. 2001; Hofmann et al. 2003). Most importantly, the SCNT technique was historically the most important advance in porcine genome engineering (Polejaeva et al. 2000), but the procedure is massive and complex, and the efficiency is extremely low. Similar to other species, the lack of functional porcine ES cells impedes the generation of genetically-engineered pigs.

The first ZFN-mediated knockout pigs were successfully generated in 2011 (Whyte et al. 2011), after which several groups generated knockout pigs using TALEN (Carlson et al. 2012) and CRISPR/Cas9 (Hai et al. 2014). Currently, engineered endonuclease-based genome editing are actively used to develop genetically-modified pigs that recapitulate human diseases in pigs (Watanabe et al. 2013; Umeyama et al. 2016), donor pigs for xenotransplantation (Hauschild et al. 2011; Miyagawa et al. 2015), and organ-deficient pigs for the production of humanized organs by blastocyst complementation (Nagashima and Matsunari 2016), and agricultural use (Rao et al. 2016; Whitworth et al. 2016).

## Non-human primate

Non-human primates (NHPs) are the ultimate animal models (phylogenetically closest to humans) that have been used in the fields of studying psychiatric disorders (Bachevalier et al. 2001; Yang et al. 2008; Gilley et al. 2011), metabolic function (O’Sullivan et al. 2013), reproductive biology (Wolf 2009; Kundu et al. 2013), and immunology (Thomas et al. 1982; Gallo et al. 1989), as those conditions cannot always be recapitulated by the rodent model. In spite of the cognitive and psychological superiority of NHPs to the other species, there are some limitations in using NHPs as an animal model such as ethical concerns when using higher primates, supply limitations, and relative cost ineffectiveness compared to smaller mammals, which should be of concern in the development of genetically-modified NHPs (Chan 2013).

Along with the NHP-specific limitations above, former technologies established in other species such as rodents were inefficient for manipulating the genome in monkeys, leading to rather slow progress regarding the generation of transgenic NHPs. In 2014, rhesus and cynomolgus monkeys were the first to be genetically modified with CRISPR/Cas9 (Niu et al. 2014) and TALEN (Liu et al. 2014), followed by genetically edited NHPs using ZFN in 2016 (Sato et al. 2016), and several subsequent NHP models that used engineered endonucleases have been successfully developed, opening a new era of genetic engineering in NHPs.

## Strategies for precise laboratory animal model mimicking human diseases

Till now, we mainly described the studies achieving in-del mutations with engineered endonucleases. However, precise genome editing harnessing the power of HDR must be the main goal regardless of the species. Using this mechanism, animal models mimicking disease-associated single nucleotide polymorphisms (SNPs) can be modeled in diverse animal species. Furthermore, considering the future use of engineered endonucleases as a drug for a gene therapy, sequence-humanized animal models will be produced and may be an essential system for preclinical studies evaluating drug safety and effectiveness. The specific knock-in (KI) strategy that uses CRISPR/Cas9 is based on the co-injection of CRISPR/Cas9 components with double-stranded DNA or single-stranded oligodeoxynucleotides (ssODN) templates, and ssODN-mediated KI in mammalian cells occurs through the HDR mechanism and is more efficient than using double-stranded donor plasmids (Radecke et al. 2010; Chen et al. 2011). The utilization of this platform enables the insertion, deletion, or replacement of genetic materials of interest into the genome (Ma et al. 2014; Shao et al. 2014). Several small molecules, such as RAD51-stimulatory compound 1 (RS-1) or Scr7, which enhance the HDR pathway or inhibit the NHEJ pathway, are known to increase the HDR efficiency when coupled with the CRISPR/Cas9 system in mammalian cells (Chu et al. 2015; Maruyama et al. 2015), mice (Maruyama et al. 2015), and rabbits (Song et al. 2016a), but difficulties remain with the KI strategy in cell lines and animals due to the low HDR frequency, which remains to be optimized to attain higher efficiency. In alignment with efforts for higher efficiency in modeling SNPs, it is recently revealed that RNA-guided deaminases including adenine base editors (ABEs) and cytosine base editors (CBEs), composed of an engineered deaminase and a catalytically impaired Cas9 variant, can introduce a single-base-pair conversion A:T to G:C or vice versa at a target site without DSBs (Gaudelli et al. 2017; Komor et al. 2016), enabling efficient programmable base editing. Among these newly designed chimeric nucleases, base editor 3 (BE3), one of CBEs, was successfully adopted in generating mice with point mutation at the target site with high efficiency (Kim et al. 2017), but its application in another laboratory animals remains to be further elucidated.

## Conclusion and perspective

The initial uses of engineered endonucleases have demonstrated their possible applications for establishing novel model organisms and genome editing systems are constantly evolving. Particularly, through genome editing using these engineered endonucleases, there are tremendous efforts to recapitulate the human disease-associated mutations in various mammalian species. Precisely-designed animals to mimic the patient-derived mutations can be more translational and thus narrow down the gaps currently present between preclinical and clinical studies.

However, the production of mutant animals with precisely-defined genetic alterations is still challenging. To enhance the efficiency of HDR in diverse mammalian species, we need to study the specific physiology involved in the regulation of HDR in fertilized eggs. There are many suggestions possibly beneficial to increase the genome-editing efficiency (e.g., suppressing NHEJ or enhancing HDR), but their exact underlying mechanisms and appropriate strategies of applications for various mammals remain to be elucidated. Furthermore, each model mammals show species-specific reproductive characteristics such as estrous cycle and gamete physiology, which may affect the HDR in fertilized eggs and thus should be taken into account for mammalian genome editing.

Taken together, the use of genetically-modified mammals generated with engineered endonucleases can provide both fundamental and advanced model system or platform to understand physiological and pathological phenomena and thus help us to develop new drugs and treatments for human diseases.
